# Progress in Neurology

**Published:** 1901-07-13

**Authors:** 


### PROGRESS IN NEUROLOGY.

(*Continued from page* 233.)

**Chronic Arsenical Poisoning.**—The symptoms of chronic arsenical poisoning are divided by Brouardel[4] into four stages—the stage of digestive disturbances, the stage of eruptions and of laryngeal and bronchial catarrh, that of sensory disturbances, and that of paralysis. The third stage is characterised by numbness, tingling, formication, and other paræsthesiæ of the extremities, by spontaneous pain with frequent headache and extreme tenderness of the limbs. Whilst in the fourth stage there is first weakness and later actual paralysis of the extensor muscles of the toes, the dorsiflexors of the ankles, and the extensor muscles of the wrist and fingers. This paralysis, though usually limited to the extremities, may, in severe cases, invade the trunk.

As in the case of lead, it is uncertain whether the toxic substance in the blood acts primarily on the peripheral nerves or on the cells in the anterior cornua of the spinal cord, or whether it acts simultaneously on both. It is probable that the whole neuron is extremely sensitive to the toxic influence of arsenic.

The amount of arsenic required to produce toxic symptoms is governed by many factors, such as the age of the person taking it, the form in which it is taken, and the condition of the stomach when it is taken by the mouth. The smallest fatal dose hitherto recorded is 2 grs. of arsenious acid. But it is a matter of everyday experience that slight toxic symptoms, such as watering of the eyes and a silvery tongue, are produced by the medicinal use of Fowler's solution in medium doses. The activity of arsenic in solution is much greater than in the solid form. And in conjunction with another neuritic poison, such as alcohol, the drug is still more virulent. If taken into the body arsenic rapidly begins to be eliminated, but its complete elimination is a matter of some considerable time. The variations of the individual in regard to susceptibility are almost, if not quite, as marked in the case of arsenic as in that of lead. Thus where epileptics have been given a small dose of arsenic with their bromide one patient may develop a bronzed pigmentation from doses taken by another with no obvious toxic effect.

**Lead Poisoning.**—Armitage,[5] of Wolverhampton, has had much success in the treatment of inveterate cases of lead poisoning by electricity. The apparatus consisted of an ordinary large porcelain bath carefully insulated. The electrodes were of carbon, and the strength of current was about 120 milliamperes. Of course only a small proportion of this passed through the patient who was immersed in the bath. The actual strength of the current was varied to suit the individual patient; the best results were obtained when the electric cnrrent reddened the skin without causing actual pain. The time of immersion was about 15 or 20 minutes. The results were generally excellent. The principal conditions dealt with were wrist-drop, often absolute, and ankle-drop. Lead gout in the various joints was a frequent condition, also tremors. Anæmia and weak action of the heart occurred in a great many cases as a complication. Of 40 cases, 37 were cured either absolutely or partially. Not only are cases of wrist-drop and joint affections very much improved, but some of the most rapid and successful cures have been in those who have had severe colic of long standing, and heart failure.

**Degeneration of the Spinal Cord.**—Putman and Taylor[6] report five cases of diffuse degeneration of the spinal cord, very similar to those described recently by Russell, Batten and Collier under the title of subacute combined degeneration of the spinal cord. They say that the features common to the general group of lesions are a diffuse degeneration, for the most part limited to the cord, with a constant involvement of the dorsal and lateral columns. The motor and sensory roots and the peripheral nerves are normal, as is also the grey matter. The vessel changes are insignificant. The objective appearance of the vessels gives no basis for the assumption that the extensive degeneration is due to vascular causes. Again there is no evidence that it is caused by any kind of myelitis, either acute or chronic. The relatively constant distribution of the lesions in the cord depends on the anatomical arrangement of the pial blood vessels. This in no way implies disease of the vessel wall with thrombosis, but that by the vessels some cause, probably toxic, reaches the cord. Anæmic states have been shown at times to be a concomitant condition, but not necessarily a cause.

**Myasthenia gravis.**—Guthrie[7] records a fatal case of myasthenia gravis and discusses its differential diagnosis from hysteria, ordinary bulbar paralysis, and diphtheritic paralysis. Myasthenia gravis is characterised clinically by (1) bulbar paralysis, usually slight, but at times extreme, affecting the lips, tongue, soft palate, pharynx and larynx; (2) weakness of the upper facial muscles, especially of the extrinsic ocular group, causing ptosis, ophthalmoplegia, or inability to close the eyes. The midfacial muscles, *e.g.*, zygomatic, seem commonly to escape. Severe or fatal attacks of dyspnœa may be due to falling back of the tongue, to abductor paralysis of the larynx, to paralysis of the intercostals or diaphragm, or to actual obstruction from food becoming impacted in the larynx; (3) weakness and readily induced fatigue of the skeletal and trunk muscles; (4) presence of the myasthenic electrical reaction (*i.e.*, the reaction of muscles to faradism, normal at first but speedily exhausted); (5) absence of muscular wasting; (6) absence of sensory affection, mental disturbance, and incompetence of sphincters; (7) remissions and exacerbations of the symptoms, both bulbar and general, are characteristic features. The paresis may disappear entirely after rest, but return on exertion. The mode of onset is usually gradual. There are no limits as to age of onset; cases beginning as early as two and a half years and as late as fifty-five.

The symptoms of hysteria may superficially resemble those of myasthenia gravis, so that at least one case of fatal myasthenia has been diagnosed as hysteria. Weakness, lassitude, and readily induced fatigue are common enough in hysteria and neurasthenia, but the presence of the electrical myasthenic reaction is absolutely characteristic. In myasthenia sensation is invariably normal. The ptosis of hysteria is due to spasm of the orbicularis and not to weakness of the levator palpebræ. Again, the dysphagia of hysteria is characterised by prodigious though unavailing efforts to swallow, whereas in myasthenia gravis there is no over-action or inco-ordination but obvious weakness of the muscles of deglutition. The speech defects are different in the two diseases; the voice in myasthenia has a marked nasal twang from paresis of the soft palate which is never met with in hysteria. The voice in myasthenia may be reduced to a whisper, but this comes on gradually whilst speaking, whereas in hysteria articulation is perfect, and if aphonia exist it does not gradually develop from the effort of speaking.

Myasthenia gravis is distinguished from ordinary bulbar paralysis by the involvement of the upper as well as the lower half of the face, by the affection of the limbs and trunk, by the myasthenic reaction, and by the variations in the severity of the symptoms. In pseudo-bulbar paralysis the limbs may be affected but their condition is one of diplegia with increased tendon jerks and rigidity.

Myasthenia gravis is perhaps more likely to be mistaken for diphtheritic paralysis than for any other disease, for in the latter affection a nasal voice, regurgitation of fluids through the nose, paralysis of the extrinsic and intrinsic ocular muscles, of the pharynx and glottis, of the intercostals and diaphragm, are exceedingly common. Again, general muscular prostration and easily induced fatigue are observed in diphtheritic paralysis. The points of distinction are the presence of the myasthenic reaction and of the knee jerks in myasthenia, whereas they are absent in diphteritic paralysis. The history of a previous sore throat, if it can be obtained, is also important. Again, diphtheritic paralysis rarely lasts beyond a few weeks, during which the symptoms are fairly constant, whereas in myasthenia gravis the symptoms last much longer and undergo variations in intensity.

[4] Brit. Med. Jour., Jan. 5 1901. [5] Birming. Med. Jour., Feb. 1901. [6] Jour. of Nerv. and Ment. Dis., Feb. 1901. [7] Lancet, Feb 9, 1901.

